# Very Late‐Onset Cytomegalovirus Disease With *Pneumocystis jirovecii* Pneumonia Coinfection in a Renal Transplant Recipient: A Case Report and Narrative Review of Published Cases

**DOI:** 10.1155/crdi/6503116

**Published:** 2026-10-28

**Authors:** Christian Wang, Susanne O. Ajao, Pinki J. Bhatt

**Affiliations:** ^1^ Department of Medicine Rutgers Robert Wood Johnson Medical School New Brunswick New Jersey USA rutgers.edu; ^2^ Division of Allergy, Immunology, and Infectious Diseases Department of Medicine Rutgers Robert Wood Johnson Medical School New Brunswick New Jersey USA rutgers.edu

**Keywords:** CMV retinitis, cytomegalovirus disease, gastrointestinal CMV disease, immunocompromised, kidney transplantation, *Pneumocystis jirovecii* pneumonia, renal transplant

## Abstract

**Background:**

Cytomegalovirus (CMV) infection is a common and serious complication following renal transplantation. It is associated with significant morbidity and mortality. The introduction of preventive measures with antiviral prophylaxis has reduced the incidence in the immediate posttransplant period, but CMV infection continues to be a prominent complication, especially once antiviral prophylaxis is discontinued. A particularly rare entity is “very late‐onset” CMV disease, which we defined as occurring 10 or more years after transplantation.

**Methods:**

We report a case of very late‐onset CMV disease presenting 17 years post–renal transplant, complicated by concurrent *Pneumocystis jirovecii* pneumonia (PJP). We performed a focused narrative review using PubMed for cases of very late‐onset CMV disease to document potential risk factors for very late‐onset CMV disease.

**Results:**

Our narrative review did not show a consistent pattern between the traditional risk factors such as prior rejection, impaired renal function, or changes in immunosuppressive therapy and the development of very late‐onset CMV disease. However, an absolute lymphocyte count below 610/μL was present in our patient and may represent an area for further investigation as a potential risk factor for very late‐onset CMV disease.

**Conclusion:**

Early recognition is crucial, as complications such as CMV–PJP coinfection carry high mortality rates and adversely affect long‐term graft and patient outcomes. Clinicians should maintain a high index of suspicion for CMV in transplant recipients regardless of the time elapsed since transplantation.

## 1. Introduction

Despite decades of experience managing cytomegalovirus (CMV) in renal transplant (RT) recipients, it remains a feared complication due to its strong association with allograft failure and increased mortality [1–3]. Per consensus definitions, CMV infection refers to the detection of CMV in peripheral blood in the absence of organ involvement, while CMV disease requires detection of CMV in tissue from the relevant organ, the presence of clinical symptoms, and end‐organ involvement [1–4]. The incidence of CMV varies widely based on multiple risk factors, the most important being donor and recipient CMV serostatus. The highest‐risk group in solid‐organ transplant (SOT) recipients is CMV‐seronegative recipients of grafts from CMV‐seropositive donors (D+/R−) [5]. To mitigate this risk, most transplant centers implement preventative strategies, with prophylactic antiviral therapy during the early posttransplant period, when immunosuppression is most intense, being the most widely used approach [6]. This strategy has changed the epidemiology of CMV disease in this population, giving rise to the entity of “late‐onset CMV disease,” defined as disease occurring after cessation of antiviral prophylaxis, typically 3–6 months posttransplantation [7]. Most late‐onset CMV disease cases arise within the first year, with risk factors including D+/R− serostatus, impaired renal function (eGFR < 45 mL/min/1.73 m^2^), and receipt of antilymphocyte therapy [8]. Rarely, however, CMV disease has been reported more than a decade after transplantation, termed “very late‐onset CMV disease” [9]. Here, we describe a case of very late‐onset CMV disease occurring 17 years post‐RT in a patient without a documented recent escalation in immunosuppression or other identifiable triggers in available records.

## 2. Case Presentation

A 41‐year‐old male with a history of end‐stage renal disease secondary to reflux nephropathy underwent a living donor RT in India in 2005 (donor CMV serostatus unknown; recipient seropositive). He immigrated to the United States in 2015 and had no documented prior rejection episodes or need for increased immunosuppression. In 2022, he was transferred to our institution with complaints of diarrhea, weight loss, and decreased appetite. He had been hospitalized at a different hospital for 13 days with similar complaints, including failure to thrive. His symptoms included a 1‐year history of dysphagia, unintentional weight loss of 40 lbs, and new‐onset nonbloody diarrhea. Five months earlier, an outpatient esophagogastroduodenoscopy (EGD) performed for dysphagia revealed esophageal ulcers, though pathology was nondiagnostic.

On his initial admission to the previous hospital, laboratory evaluation was significant for leukopenia and anemia (Table 1). Computed tomography (CT) of his abdomen and pelvis with contrast revealed colonic and small bowel distension. On hospital day 2, a percutaneous gastrostomy tube was placed for nutrition due to persistent dysphagia. Repeat EGD at that time showed progression of the esophageal ulcers, though biopsies were not obtained. He developed a new cough, which prompted a CT chest without contrast that revealed multifocal pneumonitis. Serum CMV PCR returned elevated at 4490 IU/mL. Due to an elevated serum CMV level, pneumonitis on CT chest, and esophageal ulcers on endoscopy, a presumptive diagnosis of tissue‐invasive CMV disease was made. On hospital day 9, he was started on a renally adjusted induction regimen of intravenous ganciclovir 2.5 mg/kg every 24 h. His baseline immunosuppressants, to which he was adherent to, included mycophenolate mofetil 360 mg twice daily, tacrolimus 1 mg twice daily, and prednisone 5 mg daily. He was not receiving *Pneumocystis jirovecii* prophylaxis prior to transfer to our institution. In the setting of presumptive CMV disease, mycophenolate mofetil was discontinued. His pancytopenia improved with granulocyte colony‐stimulating factor. On hospital day 14, he was transferred to our facility for a higher level of care.

**TABLE 1:** Laboratory parameters during hospitalization days 1 and 14.

	References	Hospital day 1	Hospital day 14
WBC (× 10^3^ μL)	4–10	3.7	10.2
Absolute lymphocyte count (× 10^3^ μL)	0.95–3.07	0.037	0.61
Hemoglobin (g/dL)	13.2–16.6	6.6	8.1
Platelet (μL)	140–440	333	276
BUN (mg/dL)	6–23	45	36
Creatinine (mg/dL)	0.5–1.2	1.73	1.6
Alkaline phosphatase (IU/L)	45–115	37	81
AST (IU/L)	10–55	8	24
ALT (IU/L)	9–46	5	39
Beta‐(1,3)‐D‐glucan (pg/mL)	< 60		> 500
Serum adenovirus PCR			Undetectable
Serum BK virus PCR			Undetectable
HIV antigen/antibody			Negative
Serum CMV IgM			Negative
Serum CMV IgG			Positive

*Note:* CMV, cytomegalovirus, BK virus, humanpolyoma virus 1.

Abbreviations: HIV, human immunodeficiency virus; PCR, polymerase chain reaction.

On arrival, the patient was cachectic with visible ribs and temporal wasting. He was comfortable and in no acute distress. Vital signs revealed a blood pressure of 88/64 mm Hg, pulse of 98 beats per minute, temperature of 98.3° F, respiratory rate of 18 breaths per minute, and oxygen saturation of 100% on room air. Physical examination was otherwise normal, with no oral ulcers noted. His laboratory values on admission are summarized in Table 1.

A repeat CT chest without contrast on total hospital day 14 (the day of transfer to our institution) showed patchy ground‐glass consolidation predominantly involving the middle and upper lung lobes with sparing of the bases (Figure 1a,b). Given the atypical distribution for CMV pneumonitis, bronchoscopy was planned. By total hospital day 17, the patient developed new hypoxemia requiring 2 L/min supplemental oxygen by nasal cannula. Additional workup revealed an elevated serum beta‐(1,3)‐D‐glucan (> 500 pg/mL), prompting the empiric initiation of intravenous trimethoprim–sulfamethoxazole 15 mg/kg/day with adjunctive corticosteroids on hospital day 20 for suspected *Pneumocystis jirovecii* pneumonia (PJP). Bronchoscopy with BAL confirmed the diagnosis, showing *Pneumocystis jirovecii* cyst forms on cytology fungal stain and positive *Pneumocystis jirovecii* PCR in the BAL sample, while immunohistochemical staining for CMV was negative.

**FIGURE 1:**
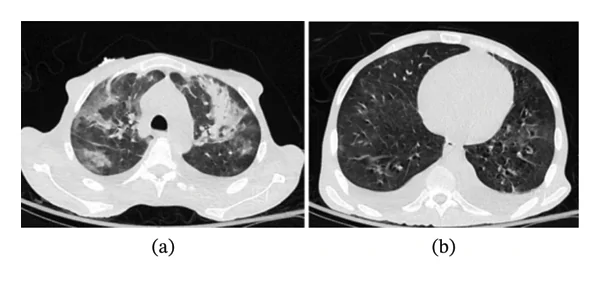
(a) Axial CT chest without contrast showing patchy areas of ground‐glass opacities and consolidation primarily in the upper and middle lung zones. This distribution was considered atypical for CMV pneumonitis, which led to bronchoscopy and PJP diagnosis. (b) CT chest without contrast. Patchy areas of ground‐glass opacities and consolidation showing sparing of the lung bases.

His course was further complicated by left‐sided CMV retinitis, requiring intravitreal foscarnet injections, followed by left‐sided retinal detachment necessitating surgical repair. He also developed *Pseudomonas aeruginosa* bloodstream infection as a result of a complicated urinary tract infection, likely related to prior Foley catheterization, and *Enterococcus faecalis* catheter‐related bloodstream infection; both resolved with appropriate antimicrobial therapy and catheter removal. Although his diarrhea initially improved, it recurred with hematochezia requiring multiple blood transfusions. He progressed to hemorrhagic shock as a complication of gastrointestinal bleeding. He was upgraded to the intensive care unit, where he was intubated for airway protection. Endoscopy and colonoscopy on total hospital day 29 revealed multiple nonbleeding ulcers in the esophagus, stomach, ascending, descending, and sigmoid colon (Figure 2). Biopsies of the ascending and descending colonic ulcers were positive for CMV on immunohistochemical staining, confirming tissue‐invasive gastrointestinal disease. Due to colonic perforation with ongoing gastrointestinal bleeding, he underwent a partial colectomy with end‐ileostomy.

**FIGURE 2:**
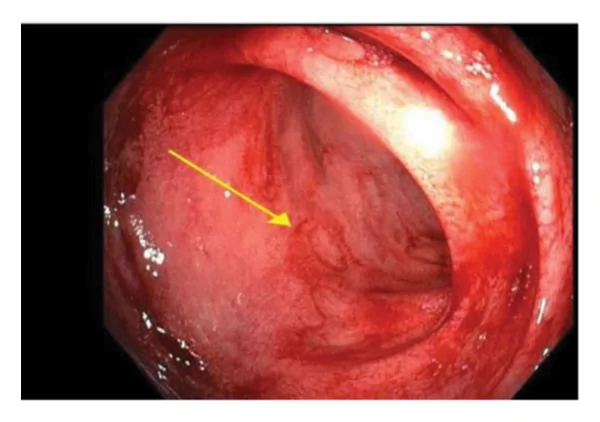
Colonoscopy: Five 4‐ to 7‐mm shallow nonbleeding ulcers were found in the descending and sigmoid colon (yellow arrow). Biopsies of colonic ulcers were found positive for CMV on immunohistochemical staining, confirming tissue‐invasive gastrointestinal CMV disease.

After completing a 21‐day treatment course of trimethoprim–sulfamethoxazole, he transitioned to secondary prophylaxis with atovaquone 1500 mg daily. Trimethoprim–sulfamethoxazole was discontinued due to pancytopenia. At the time of writing, he remains on this regimen. Intravenous ganciclovir was continued until he had consecutive undetectable CMV levels for 2 weeks (Table 2). He clinically improved and was discharged after a 61‐day hospitalization. Given the severity and extent of his disease, valganciclovir prophylactic therapy was continued for approximately one year, at which point it was discontinued once CMV PCR remained undetectable and his absolute lymphocyte count (ALC) remained above 610/μL. At his most recent follow‐up, at the time of this writing, he remained clinically well.

**TABLE 2:** Trend of serum CMV PCR values.

Hospital day	CMV PCR (IU/mL)
Day 8	4490
Day 17	922
Day 24	684
Day 31	474
Day 38	145
Day 45	< 35
Day 51	< 35

*Note:* CMV PCR lower level of detection is 35 IU/mL. CMV, cytomegalovirus.

Abbreviation: PCR, polymerase chain reaction.

## 3. Methods

We performed a focused narrative review of published cases of very late‐onset CMV disease in adult RT patients. PubMed was searched between February 1, 2025, and February 1, 2026, using the following string: (“cytomegalovirus” [MeSH Terms] OR cytomegalovirus [Title/Abstract] OR CMV[Title/Abstract]) AND (“kidney transplantation” [MeSH Terms] OR renal transplant [Title/Abstract] OR kidney transplant [Title/Abstract]) AND (“very late‐onset” [Title/Abstract] OR “very late onset” [Title/Abstract] OR “late‐onset” [Title/Abstract] OR “late onset” [Title/Abstract]). Sources were filtered by case reports. From this initial search, 28 sources were identified and screened. OpenEvidence, an AI‐based clinical literature retrieval tool, was used to identify candidate references and was solely used as a reference discovery tool to assist in identifying additional cases that were then retrieved from primary publication in PubMed. The database search was supplemented by reviewing reference lists of all included reports from the initial PubMed search to include all relevant cases. We included case reports or case series that described adult (≥ 18 years) RT recipients with CMV disease occurring ≥ 10 years after transplantation. Cases were excluded if they described CMV viremia without signs of end‐organ disease, involved nonrenal solid organ transplants, or described cases in which CMV disease occurred less than 10 years posttransplantation, with the caveat of one borderline case that was retained as its onset was within 1 year of the 10‐year threshold. Based on the aforementioned criteria, 8 sources were included in this review. For each eligible case, we extracted and listed in Table 3 the patient’s age, sex, donor and recipient CMV serostatus, history of prior CMV disease, time from transplantation to CMV disease onset, history of prior rejection episodes, kidney function at time of CMV disease, and maintenance immunosuppressive regimen.

**TABLE 3:** Summary table of 8 published sources of very late‐onset CMV disease, defined as onset of CMV disease ≥ 10 years after renal transplantation.

	Age	Gender	CMV status	Prior CMV disease	Years after Tx	Prior rejection	Kidney function	Immunosuppression	Notes
Case 1 Khan 2021 [9]	47	M	D+/R−	No	18 years	No	Cr 3.33 mg/dL	Pred, MMF	Case series
Case 2 Khan 2021 [9]	68	M	D+/R+	No	15 years	No	Cr 1.92 mg/dL	Pred, MMF	Case series
Boobes 2004 [10]	57	M	Unknown/R+	Not reported	12 years	Yes	Cr 2.26–2.49 mg/dL	Cyclo, MMF, Pred	Case report
Slifkin 2001 [11]	45	F	D−/R−	No	22 years	No	Cr < 1.0 mg/dL	Aza	Case report
Burgan 2017 [12]	69	M	D−/R−	No	12 years	5 years prior	eGFR 15 mL/min/1.73 m^2^	Cyclo, MMF, Pred	Case report
Our case	41	M	Unknown/R+	No	17 years	No	Cr 1.73 mg/dL	Tacro, MMF, Pred	Case report
Mok 2025 [13]	64	F	Unknown	No	21 years	No	Cr 2.83 mg/dL	Tacro, MMF, Pred	Case report
Gallego 2018 [14]	65	M	Unknown/R+	Not reported	24 years	History of acute rejection	Cr 1.9–2.1 mg/dL	Cyclo, MMF, Pred	Case report
^∗^Iwai 2020 [15] Case 1	69	M	Unknown/R+	Yes within 1 year of tx	9 years 9 months	Acute rejection within 1 year of transplant	Cr 1.5 mg/dL	Tacro, MMF, Methylpred	Case series
Iwai 2020 [15] Case 2	42	F	Unknown/R+	Yes within 1 year of tx	15 years	Acute rejection within 1 year of transplant	Cr 3.8 mg/dL	Tacro, MMF, Pred	Case series
Iwai 2020 [15] Case 3	71	M	Unknown/R+	Yes within 1 year of tx	13 years and 5 months	Not reported	Cr 1.3 mg/dL	Cyclo, MMF, Pred	Case series
Soto 2007 [16]	44	F	Unknown	Not reported	13 years	Not reported	Cr 1.5 mg/dL	Cyclo, Aza, Pred	Case report

*Note:* D, donor; R, recipient; CMV, cytomegalovirus; Tx, transplant; Cr, creatinine (49–104 μmol/L and 0.55–1.18 mg/dL); Pred, prednisone/prednisolone; MMF, mycophenolate; Aza, azathioprine; Cyclo, cyclosporine; Tacro, tacrolimus, Methylpred, methylprednisolone.

^∗^Case included despite onset marginally below the 10‐year threshold (Iwai Case 1: 9 years 9 months), as disease onset occurred within 3 months of the cutoff; it was deemed sufficiently representative of very late‐onset CMV disease to warrant inclusion.

## 4. Discussion

This paper reports a rare case of very late‐onset CMV disease occurring more than a decade after renal transplantation, complicated by concurrent PJP, in a patient without typical CMV risk factors. CMV disease is a well‐recognized complication of solid‐organ transplantation, as the immunosuppression needed to prevent rejection also predisposes patients to CMV reactivation [17]. Most cases occur within the first 3 months following transplant, when immunosuppressive therapy is most intense [18]. To mitigate this risk, antiviral prophylaxis is routinely administered during the early posttransplant period. While effective in reducing early CMV disease, this strategy has shifted the epidemiology, with more cases now emerging after prophylaxis is discontinued [7].

In a retrospective cohort study conducted by Santos et al., 15,848 adult RT recipients were evaluated, comparing CMV disease occurring within 100 days of transplantation with disease occurring after 100 days. The investigators found that 1.2% of RT recipients developed CMV disease within 100 days posttransplant, while 4% developed what they termed delayed‐onset CMV disease, manifesting beyond 100 days posttransplant [7]. The timing of delayed‐onset CMV, however, varies widely. In a separate study by Browne et al. that followed 2489 RT recipients between 1985 and 2007, 380 developed CMV disease, of whom 77 (20%) were classified as having late CMV infection, defined as occurring 1 year or more posttransplant. Among these, 54 cases occurred within five years, 15 between 6 and 10 years, four between 11 and 15 years, and four more than 15 years after transplantation [2]. This heterogeneity in timing has led to the term very late‐onset CMV infection to describe cases occurring ten or more years following transplantation. The patient described in our case developed CMV disease 17 years after his transplantation.

Very late‐onset CMV disease is rarely reported in the literature, and affected patients are often stable without the typical risk factors seen in delayed‐onset CMV disease. In contrast, delayed‐onset CMV disease, defined as occurring more than 100 days posttransplant, most commonly develops following discontinuation of CMV antiviral prophylaxis and has been associated with donor/recipient CMV serostatus (particularly D+/R−), intensified immunosuppression or use of antilymphocyte therapy, impaired renal function, and prior rejection episodes [5, 19]. Our patient did not demonstrate many of these risk factors; however, this observation should be interpreted with the caveat that donor CMV status was unknown.

One documented risk factor for CMV disease that was present in our case was an ALC below 610. This observation prompted us to perform a literature review to identify other potential risk factors for very late‐onset CMV disease in RT patients. Recognition of these factors could enhance monitoring strategies, support earlier diagnosis, and inform prevention efforts for this uncommon but clinically significant presentation.

Earlier tissue diagnosis was delayed in our patient as repeat endoscopy during his first hospitalization demonstrated progression of his esophageal ulcers without biopsies being obtained. This represented a missed opportunity for an earlier diagnosis and treatment. The etiology of esophageal ulcerations on endoscopy has a broad differential including CMV, herpes simplex virus, reflux, and pill‐induced [20, 21].

Table 3 summarizes our literature review, which primarily consisted of individual case reports of very late‐onset CMV disease following renal transplantation. For this review, we defined very late‐onset CMV disease as infection occurring 10 or more years after RT and examined the presence of risk factors commonly associated with delayed CMV disease. No consistent associations were found between very late‐onset CMV disease and prior rejection episodes, impaired renal function, recent changes in immunosuppressive therapy, or prior CMV disease. A notable limitation, however, was that in many cases, either the donor, recipient, or both had unknown CMV serostatus. These findings underscore the challenge of preventing very late‐onset CMV disease, as it is inconsistently reported, making it difficult to draw conclusions on potential risk factors.

As mentioned earlier, our patient had an ALC below 610 cells/μL, which was proposed by Meesing and Razonable as a possible threshold for increased risk of CMV infection, although their study did not reach statistical significance [22]. In a later retrospective study from the Mayo Clinic, they found that RT patients who had an ALC below the threshold of 610 cells/μL were at statistically significant greater risk for CMV infection or disease [23]. Neither study focused on patients many years posttransplantation; therefore, it is unclear if this association holds true in this population. Since ALC was not consistently reported in other published cases of very late‐onset CMV disease, it could not be analyzed across the cohort. It remains unclear whether this marker has relevance in this subgroup; however, it could be an area of further investigation as a potential useful value to monitor in RT recipients.

Another important aspect highlighted by this case is the potentially fatal complication of PJP coinfection. CMV infection has been associated with an increased risk of PJP infection [24, 25]. In a study by Lee et al., RT recipients with concurrent PJP and CMV infection had increased pneumonia severity, worse graft function, and a higher incidence of graft failure compared with those who developed PJP alone [26]. The proposed mechanism based on experimental and clinical data for this association is CMV‐mediated immunomodulation through its suppression of the T‐cell proliferative response by downregulation of MHC Class I/II molecules and causing functional exhaustion of CD4+ T cells, blunting the cytokine response to bacterial, fungal, and viral infections, and lastly direct NK cell suppression [27, 28]. Collectively, CMV’s immunomodulation mechanism may create a permissive environment for opportunistic infections such as PJP as observed in our case [27, 29]. In our case, the diagnosis of PJP was delayed due to the presumptive diagnosis of CMV pneumonitis as the cause of the CT chest findings. Ultimately, PJP was diagnosed through BAL testing, and CMV pneumonitis was never clearly established. Clinicians should maintain a high index of suspicion for PJP in transplant recipients with CMV infection.

This review has several limitations. It is a narrative review; therefore, it did not incorporate a systematic design. It was obtained through a single database and was made up of individual case reports or case series, which are subject to publication bias toward more unusual cases. Reporting of these cases was also inconsistent with many risk factors missing across cases, limiting any conclusions about these risk factors.

## 5. Conclusion

Very late‐onset CMV disease is an uncommon but clinically significant complication of SOT, with most available evidence limited to case reports and small cohort studies. Consequently, there is little guidance on prevention or surveillance strategies for this population. Our case highlights the challenge of surveillance strategies, as the patient lacked the conventional risk factors for CMV yet still developed very late‐onset CMV disease. Consistent with this observation, our review of reported cases demonstrated no consistent association with traditional risk factors for very late‐onset CMV disease, underscoring the unpredictability of this entity.

Importantly, however, our patient’s ALC was below 610 cells/μL, which has been associated with CMV disease in transplant recipients and could be an area of further investigation in this population. Research on such parameters may help clinicians identify patients at risk for very late‐onset CMV disease, prompt earlier CMV testing, and reduce the morbidity associated with delayed recognition.

Furthermore, the coinfection with PJP highlights the need for heightened awareness of concurrent opportunistic infections given their association with worse outcomes. Further studies are needed to better define risk factors specific to very late‐onset CMV disease and to establish evidence‐based strategies for prevention. Until then, clinicians should maintain a high index of suspicion for CMV in transplant recipients presenting with gastrointestinal or pulmonary manifestations, regardless of the time elapsed since transplantation.

**Nomenclature**
CMV: Cytomegalovirus
PJP: *Pneumocystis jirovecii* pneumonia
RT: Renal transplant
D+/R−: CMV seropositive donor and seronegative recipient
WBC: White blood cell count
BUN: Blood urea nitrogen
AST: Aspartate aminotransferase
ALT: Alanine aminotransferase
PCR: Polymerase chain reaction

## Author Contributions

Conceptualization and visualization, Susanne O. Ajao and Pinki J. Bhatt; writing–original draft preparation and writing–review and editing, Christian Wang, Susanne O. Ajao, and Pinki J. Bhatt; supervision, Pinki J. Bhatt.

## Funding

This research received no external funding.

## Disclosure

All authors have read and agreed to the published version of the manuscript.

## Ethics Statement

Written informed consent cannot be obtained; therefore, the case was appropriately deidentified in accordance with ICMJE recommendations.

## Conflicts of Interest

The authors declare no conflicts of interest.

## Data Availability

Data sharing is not applicable to this article as no datasets were generated or analyzed during the current study.
